# Assessing an Automated Noncontrast CT–based Pipeline for Sacral Tumor Classification Using a Hip Bone Reference Frame

**DOI:** 10.1148/rycan.250098

**Published:** 2026-01-02

**Authors:** Fei Zheng, Ping Yin, Kewei Liang, Li Yang, Tao Liu, Wenjia Zhang, Yujian Wang, Wenhan Hao, Qi Hao, Nan Hong

**Affiliations:** ^1^Department of Radiology, Peking University People’s Hospital, No. 11 Xizhimen South Street, Xicheng District, Beijing 100044, PR China; ^2^Intelligent Manufacturing Research Institute, Visual 3D Medical Science and Technology Development, Fengtai District, Beijing, PR China; ^3^Department of Radiology, Shanxi Provincial People’s Hospital, Taiyuan, Shanxi, PR China

**Keywords:** Applications - CT, Deep Learning, Radiomics, Segmentation, Skeletal-Axial, Pelvis, Sacral Tumors

## Abstract

**Purpose:**

To develop a fully automated hybrid approach to predict sacral tumor types from preoperative noncontrast CT (NCCT ) images.

**Materials and Methods:**

In this retrospective, multicenter study, scans were available in 690 patients who had histopathologically confirmed preoperative sacral NCCT performed between January 2011 and May 2024. A fully automated hybrid model integrated two deep convolutional neural network models (model 1 and model 2) through a fully automated pipeline. Model 1 segments tumors and hip bones automatically from NCCT images, producing masks that are used by model 2. For the first time, the hip bone was used as a reference frame for tumor localization. The second model, CL-MedImageNet, is an innovative six-classification model that allows the simultaneous input of tumor images, clinical data, and location information. This streamlined, automated system ensures efficient data integration and processing between the two models. The efficacy of the model was assessed in comparison to that of radiologists, using metrics including the area under the curve (AUC), F1 score, and confusion matrix.

**Results:**

In all, 690 patients (mean age, 46 years ± 17 [SD]; 377 male patients) were included. Segmentation achieved mean Dice coefficients of 0.82 ± 0.11 (validation), 0.81 ± 0.12 (internal test), and 0.81 ± 0.12 (external test) after postprocessing; interobserver Dice coefficient was 0.96. The CL-MedImageNet classifier attained macro average AUCs of 0.89 (95% CI: 0.83, 0.93), 0.88 (95% CI: 0.84, 0.92), and 0.87 (95% CI: 0.79, 0.92) in validation, internal, and external test sets, respectively, with macro average F1 scores of 0.63, 0.63, and 0.56. The highest achieved precision and sensitivity were both 0.66 across all sets. CL-MedImageNet outperformed radiologists (macro average AUCs, 0.87 vs 0.80, *P* = .002; 0.87 vs 0.83, *P* = .45).

**Conclusion:**

The fully automated NCCT-based CL-MedImageNet pipeline demonstrated high segmentation accuracy and robust six-class classification, outperforming expert radiologists.

**Keywords:** Applications - CT, Deep Learning, Radiomics, Segmentation, Skeletal-Axial, Pelvis, Sacral Tumors

*Supplemental material is available for this article.*

© The Author(s) 2026. Published by the Radiological Society of North America under a CC BY 4.0 license.

See also commentary by Rouzbahani in this issue.

## Graphical abstract

**Figure:**
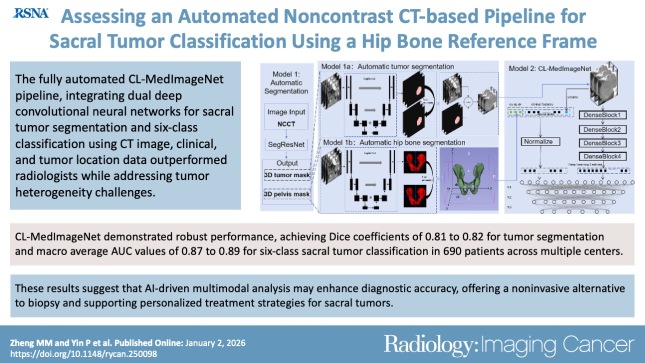

**Summary**

An automated deep learning–based classification model outperformed radiologists in noncontrast CT-based segmentation and six-class classification of sacral tumors.

**Key Points**

■ In this retrospective noncontrast CT study of 690 patients with histopathologically confirmed sacral tumors, automated segmentation achieved Dice coefficients of 0.82, 0.81, and 0.81 for validation, internal, and external test sets, respectively.
■ The fully automated deep learning–based classification model, CL-MedImageNet, yielded macro average area under the curve (AUC) values of 0.89, 0.88, and 0.87 for validation, internal, and external test cohorts, respectively, with macro average F1 scores of 0.63, 0.63, and 0.56.
■ At external testing (*n* = 60), CL-MedImageNet outperformed two radiologists (macro average AUCs: 0.87 vs 0.80, *P* = .002; 0.87 vs 0.83, *P* = .45), achieving macro average precision of 0.56 and macro average sensitivity of 0.55.

## Introduction

Bone tumors encompass a variety of types and are the third leading cause of cancer-related death in patients under 20 years old (1,2). Primary malignant bone tumors, such as osteosarcoma and chondrosarcoma (3), originate from mesenchymal cells and account for about 0.2% of all malignancies worldwide (4). Osteosarcoma, the most common primary malignant bone tumor, represents over 44% of cases (5), and its treatment relies primarily on chemotherapy and surgical resection (6,7). Chondrosarcoma, the second most common malignant bone tumor, responds poorly to chemotherapy and radiation therapy (8) and is thus treated primarily by surgical excision. Primary benign bone tumors are more difficult to quantify by incidence and are generally managed with minimally invasive procedures aimed at preserving function (9). Metastatic bone tumors pose additional challenges, constituting 15%–20% of metastatic cases (10), and are treated with drug therapy, radiation therapy, or surgery, depending on the tumor site (11,12). In clinical practice, treatments vary substantially based on tumor type (13).

Sacral tumors, a distinct subset of neoplasms, present unique diagnostic and therapeutic challenges. Sacral chordoma is most prevalent (approximately 40% of sacral tumors), followed by sacral giant cell tumor (14). Both tumors are often diagnosed late due to nonspecific early symptoms (15), and their rarity can lead to misdiagnosis, resulting in unnecessary biopsies or delayed treatment, which increases patient discomfort, costs, and mortality risks (16). The treatment strategies for sacral tumors are also distinct and vary based on the tumor type. For sacral chordoma, lumbar resection is preferred to reduce its high recurrence rate, while sacral giant cell tumor is generally treated with intralesional curettage (13). Therefore, accurate preoperative identification and differential diagnosis are crucial for personalized treatment.

Biopsy is commonly used to classify tumor histology before surgery, but it is invasive, prone to sampling errors, and may cause complications (17). In contrast, imaging has become fundamental in the era of precision oncology for bone tumor diagnosis (18). CT is typically the first choice for sacral tumor evaluation, particularly using thin-section helical acquisition with two-dimensional and three-dimensional (3D) reconstructions, which provide critical details on tumor characteristics such as calcification, ossification, and bone destruction (14,19). Although MRI, especially multiparameter MRI, offers enhanced soft tissue contrast and quantitative data, it is less effective at depicting bone destruction and is often used alongside CT (20). In addition, the limitations of MRI such as cost, scan time, and contraindications may reduce its practicality, especially in underdeveloped regions where CT remains a more accessible and efficient option (21).

Given these challenges with traditional diagnostic methods, artificial intelligence (AI)–based solutions have emerged to improve diagnosis and guide treatment. AI is a branch of computer science that focuses on solving problems by recognizing patterns in data and making data-driven decisions (22,23). Rather than being a single technology, AI encompasses a variety of computational models and algorithms, each designed to perform specific tasks based on the real-world problem at hand (24). As AI technology progresses, its application in medical imaging has become particularly notable. In the field of medical imaging, AI has catalyzed the emergence of radiomics (25,26). Convolutional neural networks (CNNs), renowned for their efficacy in image diagnostics, have demonstrated substantial potential in leveraging radiomics to forecast diagnoses, prognoses, and therapeutic outcomes for patients with cancer (12,27). One such example is CerebralDoc, an AI-driven system using a 3D CNN for vascular reconstruction in head and neck CT angiography images. This system greatly enhances efficiency by substantially reducing postprocessing time and the number of clicks compared with manual methods, while also decreasing the labor force required, demonstrating its potential to streamline workflows and reduce medical costs (28). Another example is the application of CNNs in differentiating benign from malignant parotid tumors, particularly assisting junior radiologists in achieving higher accuracy and better clinical decision-making through model-assisted support. These examples highlight the ability of CNNs to improve diagnostic performance, support radiologists, and ultimately enhance clinical workflows (29). Several challenges hinder the clinical implementation of CNNs for sacral tumor prediction. If the process is not fully automated, tasks such as manual segmentation can be time-consuming, reducing clinical feasibility and introducing interrater variability. Additionally, current image-based CNNs struggle to incorporate 3D tumor location data, which is crucial for accurately predicting sacral tumor types (30). Only a few studies have explored the potential of radiomics for distinguishing between benign and malignant sacral tumors preoperatively (13,31–34). Moreover, given the rarity of bone tumors, no external validation tests have been performed across these published studies (13,31–33,35). Instead, they conducted validation solely within their internal datasets. Existing AI models for sacral tumor classification are limited by overreliance on internal validation, lack of integration between imaging and clinical data, and dependence on manual segmentation, which can lead to inconsistencies and inefficiencies. Our study addresses these issues by introducing external validation to enhance generalizability, developing an innovative tumor localization method to improve classification performance, integrating both clinical and imaging data for a more comprehensive diagnostic approach, and using a fully automated process to reduce human error and improve efficiency.

The aim of this study was to develop a fully automated hybrid approach to predict sacral tumor types from preoperative NCCT images. This approach integrates two key components: *(a)* using CNNs for automated tumor and hip bone segmentation and *(b)* using a CNN-based classifier for predicting the sacral tumor type. First, the tumor segmentation model segments tumors and calculates their bounding frame and center of mass relative to the voxel coordinate system, while the hip bone segmentation model segments the hip bone and transforms the voxel centroid into a hip bone–relative coordinate system. Second, the CNN-based classifier is used for predicting the sacral tumor type. The classification model integrates clinical, location, and imaging data to achieve six-class tumor classification. These innovations make our model more clinically applicable, accurate, and reliable for sacral tumor classification, supporting better preoperative decision-making and personalized treatment planning.

## Materials and Methods

### Study Design

**Figure 1:**
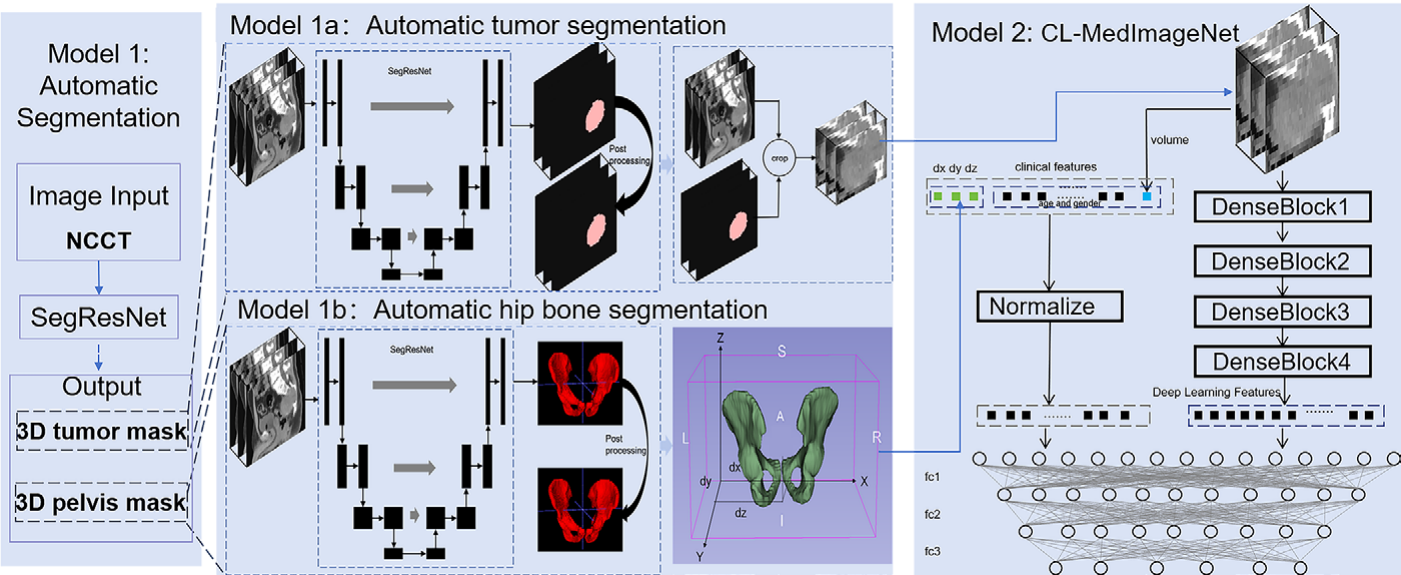
The overall flowchart of this study. We built an automated hybrid model integrating two deep convolutional neural networks (model 1 and model 2) through a fully automated pipeline. Model 1 segments tumors and hip bones automatically from noncontrast CT (NCCT) images, producing masks that are used by model 2. The hip bone was used as a reference frame for tumor localization. The second model, CL-MedImageNet, is an innovative six-classification model that allows the simultaneous input of tumor images, clinical data, and location information. 3D = three-dimensional.

This retrospective study was approved by the institutional review boards of the respective hospitals, and informed consent was waived due to its retrospective nature. The study design is outlined in Figure 1. Our automated hybrid model consists of two CNN models (model 1 and model 2) connected by a fully automated pipeline. Model 1, a CNN for automatic tumor and hip bone segmentation, was trained to generate tumor and hip bone masks from NCCT images with soft tissue window settings. Model 2, a CNN-based multiclass classifier, was trained to predict sacral tumor types based on three inputs: *(a)* cropped images from NCCT generated by model 1a, *(b)* the relative position of the tumor centroid to the hip bone from model 1b, and *(c)* clinical information including age, sex, and tumor volume. Together, these models form the hybrid system, with the fully automated pipeline facilitating seamless data flow between them.

### Patient Data and NCCT Image Acquisition

**Figure 2:**
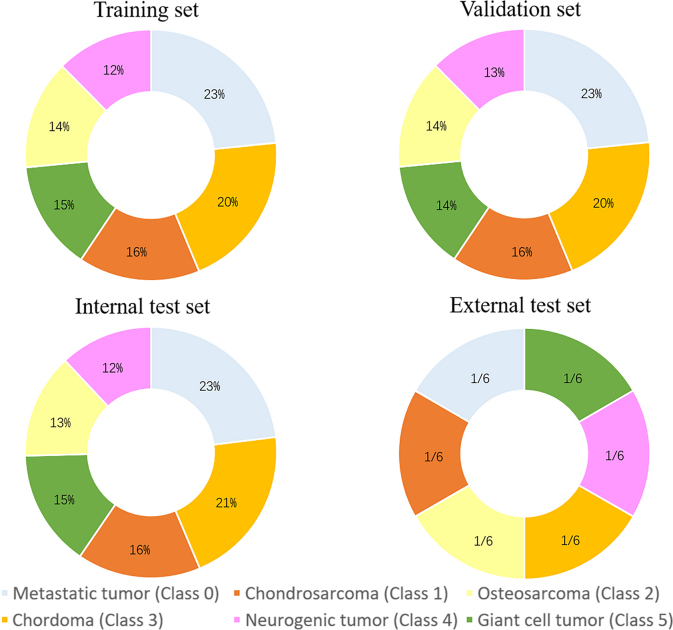
Distribution of tumor types across four datasets: training set, validation set, internal test set, and external test set. The tumor types are represented as class 0 (metastatic tumor), class 1 (chondrosarcoma), class 2 (osteosarcoma), class 3 (chordoma), class 4 (neurogenic tumor), and class 5 (giant cell tumor). The distribution is basically well-balanced across all datasets, ensuring diversity and fairness in model training and evaluation.

In this study, we collected data from three independent patient cohorts across three centers. All patients had a single sacral tumor detected on NCCT images within 1 month before their initial surgery. The primary cohort, sourced from center 1, was used for model training, hyperparameter optimization, and internal testing comprising 630 patients treated between January 2011 and May 2024. Additionally, external test cohorts were collected from centers 2 and 3, with 10 cases per category, totaling 60 patients, to serve as the external test set for final model evaluation (Fig S1). The distribution of tumor types in the training set, validation set, and test set can be seen in Figure 2 and Table S1. The study used Philips iCT 256 and GE Lightspeed VCT 64 CT scanners from Philips Healthcare and GE HealthCare, respectively. Inclusion criteria encompassed histopathologically confirmed sacral tumors, with patients having preoperative NCCT images of single sacral tumors. Exclusion criteria included prior anticancer treatment, inadequate image quality, repeated patients for follow-up or monitoring, and postoperative tumor recurrence.

All patients underwent preoperative sacral NCCT scans, with specific scanning parameters detailed in Appendix S1. The raw NCCT data, stored in Digital Imaging and Communications in Medicine (DICOM) format, were obtained from the institute’s picture archiving and communication system and were transferred to a personal computer for further processing. As part of the preprocessing, the data were first resampled to achieve voxel dimensions of 0.75 × 0.75 × 5 voxels. Since different scanning devices may use different coordinate systems, such as left-posterior-superior (LPS) or right-anterior-superior, we standardized the data by converting all coordinate systems to the unified LPS system. In addition to standardizing coordinate systems and resampling images, augmenting data represents an extra data processing procedure especially used for deep learning–assisted radiomic analyses (36,37).

### CNNs for Automatic Tumor and Hip Bone Segmentation

Of the 690 eligible patients, 180 were manually segmented and 510 automatically segmented. We created the dataset by randomly selecting 30 patients from each of the six categories in the training set of center 1, then dividing it into a training and test set with a ratio of 5:1. A radiologist with 5 years of experience in musculoskeletal imaging manually delineated the tumor regions of interest and hip bones on the NCCT images using ITK-SNAP 3.8.0 *(http://www.itksnap.org)*, layer by layer. A second radiologist with 10 years of experience verified and adjusted the accuracy of these contours. Interobserver agreement was quantified on 30 randomly selected cases by calculating the Dice coefficient.

Before automatic segmentation, various data augmentation techniques—such as cropping, flipping, rotation, intensity normalization, and noise introduction—were applied to enhance the model’s generalization ability. Detailed information on these techniques is provided in Appendix S1. Given the large image size (approximately 550 × 550 × 60 voxels), we adopted a patch-based segmentation strategy using patches sized 288 × 288 × 48 voxels, as the NVIDIA RTX A6000 GPU could not process the entire image simultaneously. The segmentation models, based on SegResNet (38), were trained on this GPU with an initial learning rate of 0.1, following a cosine decay schedule. After a brief warm-up phase, the learning rate followed a cosine decay function, rapidly decreasing in the early stages and gradually tapering off as training progressed. Although the learning rate approached zero by the end of training, it never fully reached it, ensuring smooth convergence. After all data underwent segmentation, radiologists reviewed and corrected the automatically segmented regions, mirroring the previous procedures. The postprocessing with maximum junction domain preservation was then applied to isolate the largest connected region, thereby removing minor artifacts and preserving only a single tumor or hip bone. We ultimately obtained a comprehensive dataset of tumors and hip bones.

**Figure 3:**
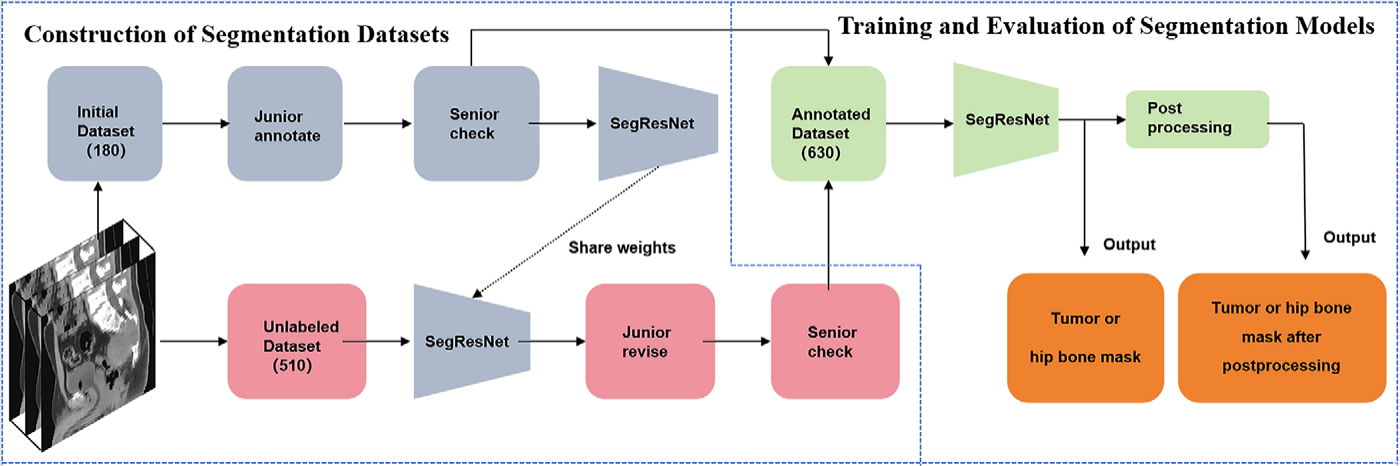
Segmentation dataset construction and model evaluation workflow. During the training and evaluation stage of segmentation, two independent models were used to segment the tumor and hip bone respectively.

Segmented data were split into ratios of 7:1:2 of training, validation, and internal test sets. The SegResNet models were trained on 200 epochs with an initial learning rate of 0.1 and cosine decay schedule after a linear warm-up. The model achieving the highest Dice score on the validation set was retained for both internal and external evaluation. Performance was reported as means ± SDs of the Dice coefficient. As shown in Figure 3, the flowchart illustrates the construction of segmentation datasets and the evaluation of the segmentation models.

### Tumor Volume and Spatial Localization

Tumor volume was calculated by counting voxels within the segmented regions of interest. A hip bone–relative coordinate system (Fig 1) was defined using the segmented hip bone: the origin was set at the intersection of the lowest (*z*-axis), leftmost (*x*-axis), and rearmost (*y*-axis) surfaces. Each axis was normalized to a 0–1 range. Tumor centroids were calculated in this coordinate system, yielding D*x*, D*y*, and D*z* distances.

### CNN for Six Classifications of Sacral Tumors

Each tumor category from center 1 was placed into training, validation, and test sets in a 7:1:2 ratio, consistent with the dataset construction method used for the segmentation model. To address the class imbalance issue, we applied class weighting adjustments by assigning higher weights to the minority classes, encouraging the model to focus more on the minority class samples during training. We adjusted the weights in the loss function during training to ensure that class imbalance did not affect the model’s learning process. The training process began with data preprocessing, where all images were resized to 256 × 256 × 64 voxels. To enhance data diversity, several augmentation techniques were applied, including random rotation (±30°), random flipping, and random zooming (scaling factor between 0.9 and 1), each with a 50% probability. These augmentations, combined with intensity normalization and noise introduction, aimed to improve the model’s generalization ability. Clinical data (age, sex, and tumor volume) and location information were standardized by removing the mean and scaling to unit variance, ensuring that all features followed a standard normal distribution, using the formula:


$$X_{scaled}=\frac{X-\mu }{\sigma }.$$

This prevents features with larger value ranges from disproportionately influencing the model. Our standardized numerical encoding was uniformly applied to all input variables (including categorical ones like sex and location) in our automated pipeline solely for computational consistency in model implementation, not for biologic rationale.

We introduce an innovative fusion network, MedImageNet, which is built upon the foundation of DenseNet. Image features were extracted using a pretrained DenseNet-121 backbone, yielding 1024-dimensional feature vectors. MedImageNet leverages deep learning features extracted from NCCT images, which are then merged with standardized clinical data and location information. The combined dataset is subsequently processed through three fully connected layers to facilitate accurate classification. The initial learning rate was set at 0.1 and followed a cosine decay schedule. Training was conducted over 100 epochs, and the best model based on validation area under the receiver operating characteristic curve (AUC) was retained. The model with the highest AUC in the validation set was chosen for subsequent internal and external testing.

In our experiment, we constructed three different six-class classification models by concatenating various feature types. First, we combined image features with clinical information to create the C-MedImageNet model. Second, we integrated image features with location information to build the L-MedImageNet model. Finally, we combined image features with both clinical and location information to develop the CL-MedImageNet model. All models followed a similar training process, using concatenated features to enhance classification performance. Additionally, we developed comparison models based on clinical, location, and imaging data. Using clinical information alone and a combination of clinical and location data, we built clinical models using a support vector machine (SVM). While kernel functions do increase the dimensionality of the feature space, our SVM model, with a sample size more than 10 times the number of variables, may be sufficiently supported for effective classification. For imaging-based models, we used DenseNet121 *(https://github.com/Project-MONAI/MONAI/blob/dev/monai/networks/nets/densenet.py)* with image features only. We applied feature engineering to address the complexity imbalance between the image and clinical models. For the image data, we used preprocessing techniques and deep learning methods to extract features, while for the clinical data, we applied standardization and normalization to ensure the features contained the maximum relevant information. These models were compared with constructed C-MedImageNet, L-MedImageNet, and CL-MedImageNet models to evaluate performance across different feature combinations.

### Fully Automated Pipeline and Hybrid Model

Our automated hybrid model was developed by integrating model 1 for segmentation with model 2 for sacral tumor type prediction through a fully automated pipeline. This hybrid model was tested on both an internal test set and an external test set, neither of which was exposed during model development. The process begins with model 1 performing automatic tumor and hip bone segmentation. From this segmentation, the tumor volume is calculated, and its relative position to the hip bone is determined. The images are cropped based on the tumor region, and they are provided as inputs for model 2, along with the tumor’s relative location and clinical data (age, sex, and tumor volume). All processes in this pipeline were fully automated. The trained models and image processing code are available at the following link: *https://github.com/Kewei-Liang/CL-MedImageNet*.

### Comparison of Radiologists’ Reading Results

To further assess the performance of our proposed model, we conducted an interpretation of radiologists’ readings of the external dataset. Two radiologists (F.Z. and Y.W.), with 5 years of experience in musculoskeletal imaging diagnosis, participated in this study. A total of 60 (60 lesions from centers 2 and 3) patients were presented to the radiologists in random order. Throughout the process, the radiologists were blinded to the diagnostic reports and final pathologic results. They were only provided with the patient’s clinical data (age, sex, and tumor volume) and the original NCCT images for diagnosis. The diagnostic results from this reading were directly compared with the results from our CL-MedImageNet model.

### Statistical Analysis

For continuous variables, analysis of variance or the Kruskal-Wallis test was applied, while categorical variables were compared using the χ^2^ test or Fisher exact test, as appropriate. All statistical tests were two-sided, and significance was defined as a *P* value less than .05. The analysis was performed using IBM SPSS Statistics (IBM). The model’s performance was assessed using the AUC and F1 score, which provided an overall measure of its discriminatory power, while confusion matrices were used to quantify the model’s accuracy. We used both AUC and F1 score to more accurately evaluate the model’s performance, particularly in predicting the minority class, while mitigating the impact of class imbalance.

## Results

### Clinical Characteristics

A total of 231 patients were excluded for the following reasons: 169 due to repeated follow-up scans, 37 for prior anticancer treatment, 16 for obvious artifacts, and nine for postoperative tumor recurrence. Cohort 1 included 630 patients (mean age, 46 years ± 17; 344 male patients). Cohorts 2 and 3 combined comprised 60 patients (mean age, 44 years ± 19; 33 male patients). The six types of tumors are classified as follows: 0 = metastatic tumor, class 1 = chondrosarcoma, class 2 = osteosarcoma, class 3 = chordoma, class 4 = neurogenic tumor, and class 5 = giant cell tumor, with detailed examples provided in Figure S2. Statistical analysis revealed substantial differences in age, sex, and the distance from the tumor centroid to the hip bone among the different sacral tumor groups. Detailed clinical characteristics of the patients are provided in Table 1.

**Table 1:** Baseline Characteristics of the Patients

Variable	Class 0	Class 1	Class 2	Class 3	Class 4	Class 5	*P* Value
Volume (cm^3^)	274.92 ± 277.56	799.62 ± 1284.57	575.45 ± 552.07	345.91 ± 410.34	464.99 ± 393.02	378.36 ± 321.93	.43
Age (y)	56.94 ± 12.50	44.48 ± 13.33	28.33 ± 13.04	58.81 ± 12.63	43.65 ± 13.68	33.69 ± 12.06	<.001
Dx (mm)	0.53 ± 0.19	0.47 ± 0.22	0.50 ± 0.23	0.50 ± 0.06	0.50 ± 0.10	0.51 ± 0.12	.66
Dy (mm)	0.65 ± 0.18	0.58 ± 0.24	0.58 ± 0.19	0.86 ± 0.14	0.65 ± 0.15	0.72 ± 0.14	<.001
Dz (mm)	0.58 ± 0.18	0.54 ± 0.22	0.60 ± 0.20	0.52 ± 0.12	0.61 ± 0.09	0.63 ± 0.16	.06
Sex							<.001
Female	68 (43.87)	60 (54.55)	42 (42.86)	41 (29.50)	48 (55.81)	60 (58.82)	
Male	87 (56.13)	50 (45.45)	56 (57.14)	98 (70.50)	38 (44.19)	42 (41.18)	

Note.—Unless otherwise indicated, data are means ± SDs for volume, age, and distances, and numbers with percentages in parentheses for sex. D*x* (distance along *x*-axis), D*y* (distance along *y*-axis), and D*z* (distance along *z*-axis), respectively, signify the coordinate positions on the *x, y*, and *z* axes. Performance comparison across tumor classes: 0 = metastatic tumor, 1 = chondrosarcoma, 2 = osteosarcoma, 3 = chordoma, 4 = neurogenic tumor, 5 = giant cell tumor.

### CNN Segmentation Performance

**Figure 4:**
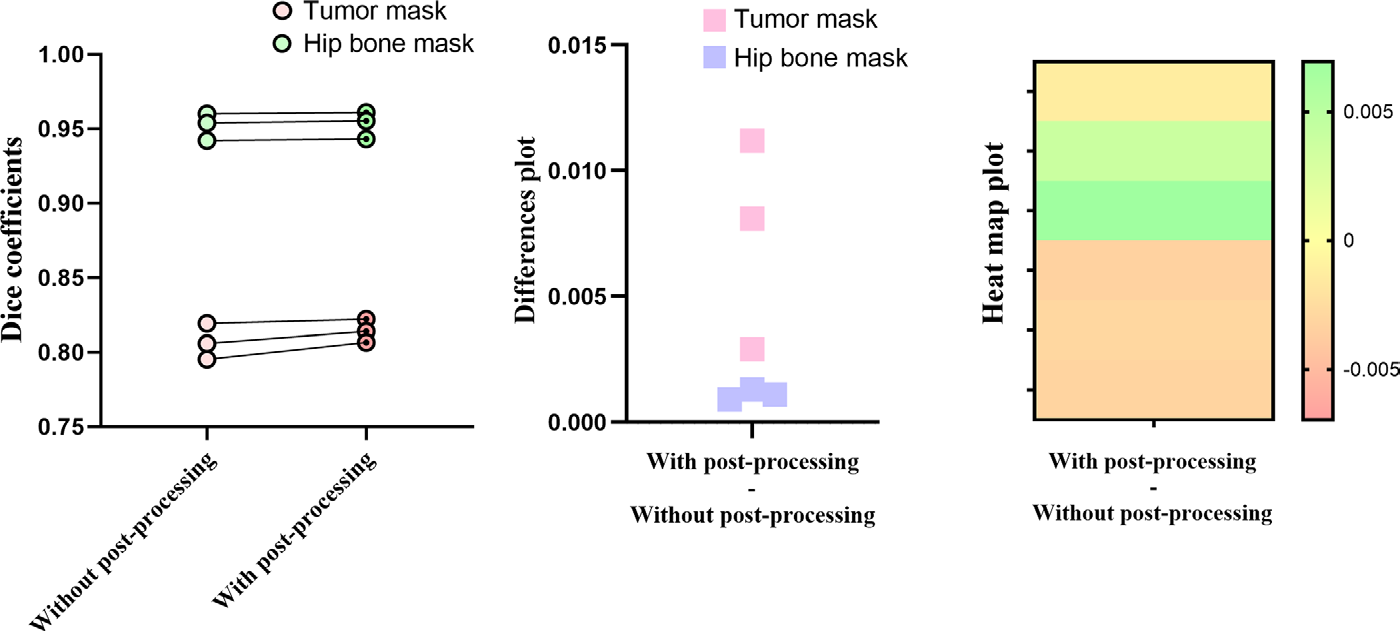
Comparison of Dice coefficients for automatic segmentations with and without postprocessing. The left panel (dot-line plot) shows the Dice coefficients for both tumor and hip bone masks before and after postprocessing, indicating an improvement in Dice values after postprocessing. The middle panel (difference plot) highlights the differences in Dice coefficients between postprocessed and non-postprocessed segmentations, with the tumor mask showing a more substantial increase in Dice values. The right panel (heatmap plot) further illustrates these differences. Overall, the results demonstrate that postprocessing enhances the accuracy of automatic segmentations, particularly for the tumor mask.

The SegResNet segmentation framework was trained for 200 epochs. Initial Dice coefficients of the validation, internal test, and external test sets were 0.82, 0.81, and 0.80, respectively. After training, the segmentation results included multiple regions, with the segmentation primarily concentrating on the vertebral endplate in addition to the tumor region. We speculated that the partial volume effect of the endplate was similar to the imaging findings of bone destruction in the tumor area. To address this, postprocessing with maximum junction domain preservation was applied to remove small artifacts and preserve a single tumor or hip bone. After postprocessing, the SegResNet model’s performance improved, with Dice coefficients of 0.82, 0.81, and 0.81 in the validation set, internal test set, and external test set, respectively, as summarized in Figure 4 and Table 2. The results of the initial automatic hip bone segmentation and the final automatic hip bone segmentation after postprocessing are also presented in Table 2. Furthermore, the Dice coefficient between the two radiologists was 0.96, indicating excellent agreement in the segmentation results.

**Table 2:** Dice Coefficients and SDs of the Initial Automatic Segmentation and the Final Automatic Segmentation after Postprocessing of Both the Tumor and Hip Bone

Mask	Validation Set	Internal Test Set	External Test Set
Tumor mask	0.82 ± 0.11	0.81 ± 0.11	0.80 ± 0.10
Tumor mask after postprocessing	0.82 ± 0.11	0.81 ± 0.12	0.81 ± 0.12
Hip bone mask	0.96 ± 0.07	0.95 ± 0.08	0.94 ± 0.09
Hip bone mask after postprocessing	0.96 ± 0.07	0.96 ± 0.08	0.94 ± 0.09

Note.—Data are mean Dice coefficients ± SDs.

### Classifications Model Performance

**Figure 5:**
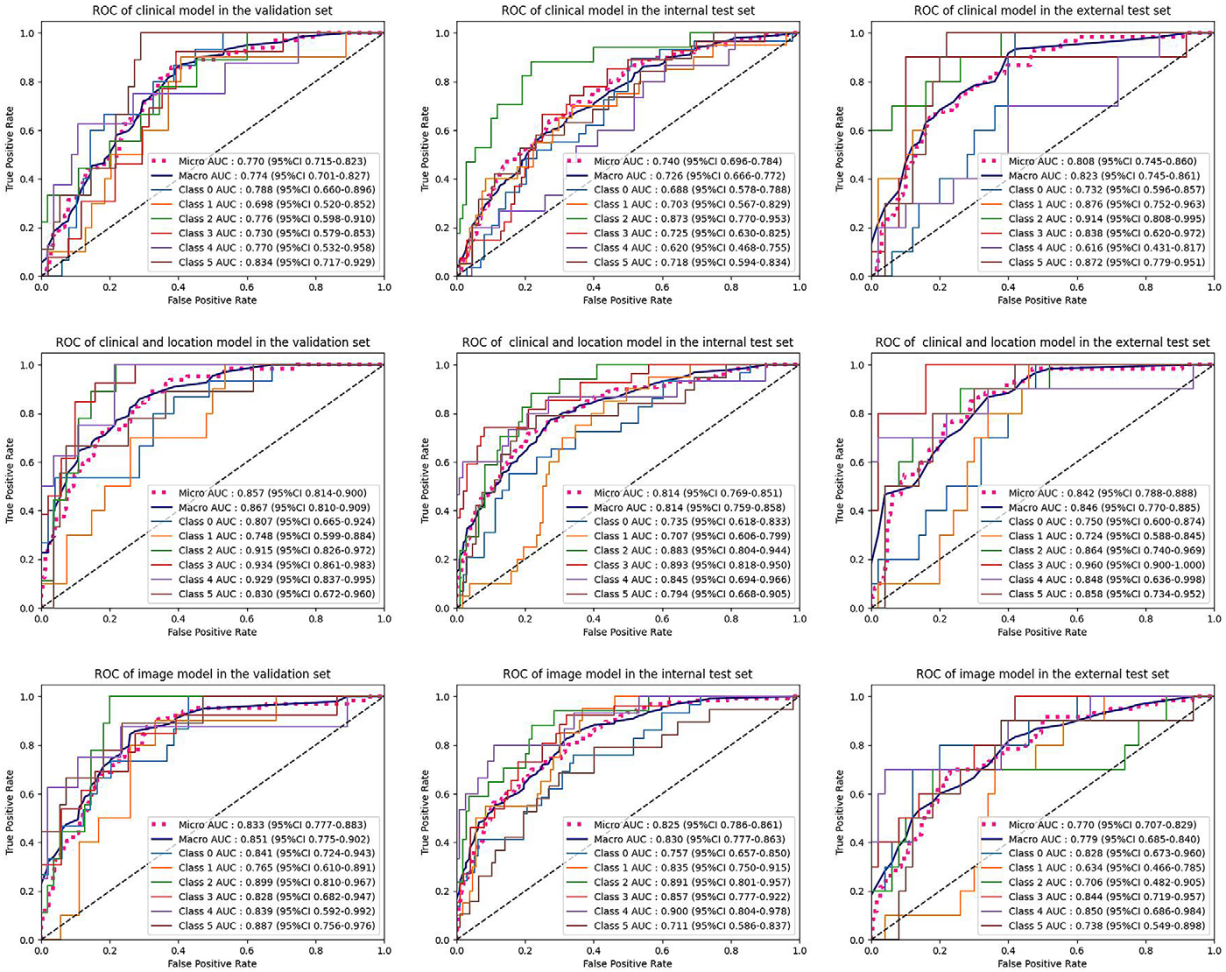
The area under the curve (AUC) values for the support vector machine (SVM) and DenseNet121 models were plotted for the validation set, internal training set, and external training set. ROC = receiver operating characteristic.

The SVM model based on clinical and location information outperformed the SVM model based solely on clinical information, achieving macro average AUC values of 0.87, 0.81, and 0.85 on the validation set, internal training set, and external training set, respectively. This suggests that location information improves the identification of different sacral tumor types. Detailed classification results for the six sacral tumor types are presented in Table 3 and Figure 5.

**Table 3:** Model Performance Comparison for Sacral Tumor Classification

Test Set	C-SVM	CL-SVM	Densnet121	C-MedImageNet	L-MedImageNet	CL-MedImageNet
Validation set						
Macro average AUC	0.774 (0.701, 0.827)	0.867 (0.810, 0.909)	0.851 (0.775, 0.902)	0.886 (0.814, 0.924)	0.885 (0.824, 0.926)	0.891 (0.831, 0.930)
Macro average F1 score	0.308 (0.235, 0.381)	0.514 (0.421, 0.598)	0.439 (0.305, 0.553)	0.61 (0.470, 0.716)	0.581 (0.449, 0.689)	0.633 (0.502, 0.745)
Micro average AUC	0.770 (0.715, 0.823)	0.857 (0.814, 0.900)	0.833 (0.777, 0.883)	0.872 (0.826, 0.916)	0.876 (0.827, 0.920)	0.875 (0.823, 0.920)
Micro average F1 score	0.344 (0.259, 0.429)	0.531 (0.436, 0.616)	0.469 (0.344, 0.594)	0.609 (0.484, 0.734)	0.578 (0.453, 0.703)	0.656 (0.531, 0.766)
*P* value	.002	.205	.264	.507	.451	
Internal test set						
Macro average AUC	0.726 (0.666, 0.772)	0.814 (0.759, 0.858)	0.830 (0.777, 0.863)	0.865 (0.817, 0.899)	0.843 (0.788, 0.884)	0.883 (0.836, 0.920)
Macro average F1 score	0.319 (0.238, 0.386)	0.504 (0.421, 0.582)	0.486 (0.384, 0.573)	0.484 (0.388, 0.571)	0.512 (0.417, 0.590)	0.632 (0.534, 0.714)
Micro average AUC	0.740 (0.696, 0.784)	0.814 (0.769, 0.851)	0.825 (0.786, 0.861)	0.862 (0.827, 0.896)	0.841 (0.805, 0.875)	0.884 (0.849, 0.915)
Micro average F1 score	0.354 (0.276, 0.441)	0.512 (0.425, 0.591)	0.492 (0.405, 0.579)	0.508 (0.421, 0.595)	0.524 (0.437, 0.611)	0.643 (0.563, 0.722)
*P* value	.006	.035	.032	.109	.144	
External test set						
Macro average AUC	0.808 (0.745, 0.860)	0.846 (0.770, 0.885)	0.779 (0.685, 0.840)	0.849 (0.775, 0.893)	0.822 (0.736, 0.878)	0.874 (0.794, 0.918)
Macro average F1 score	0.415 (0.332, 0.498)	0.483 (0.392, 0.574)	0.374 (0.257, 0.479)	0.498 (0.360, 0.611)	0.416 (0.283, 0.527)	0.555 (0.428, 0.665)
Micro average AUC	0.823 (0.745, 0.861)	0.842 (0.788, 0.888)	0.770 0.707, 0.829)	0.832 (0.786, 0.882)	0.806 (0.751, 0.861)	0.867 (0.817, 0.912)
Micro average F1 score	0.483 (0.399, 0.567)	0.467 (0.376, 0.558)	0.383 (0.267, 0.517)	0.500 (0.367, 0.617)	0.417 (0.300, 0.550)	0.550 (0.433, 0.683)
*P* value	.003	.212	.017	.291	.328	

Note.—Area under the curve (AUC) values and F1 scores with 95% CIs in parentheses for different models are used to identify various types of sacral tumors in the validation set, internal training set, and external training set. The *P* value represents the macro average AUCs’ differences between each model and the CL-MedImageNet. C = clinical information, L = location information, SVM = support vector machine.

**Figure 6:**
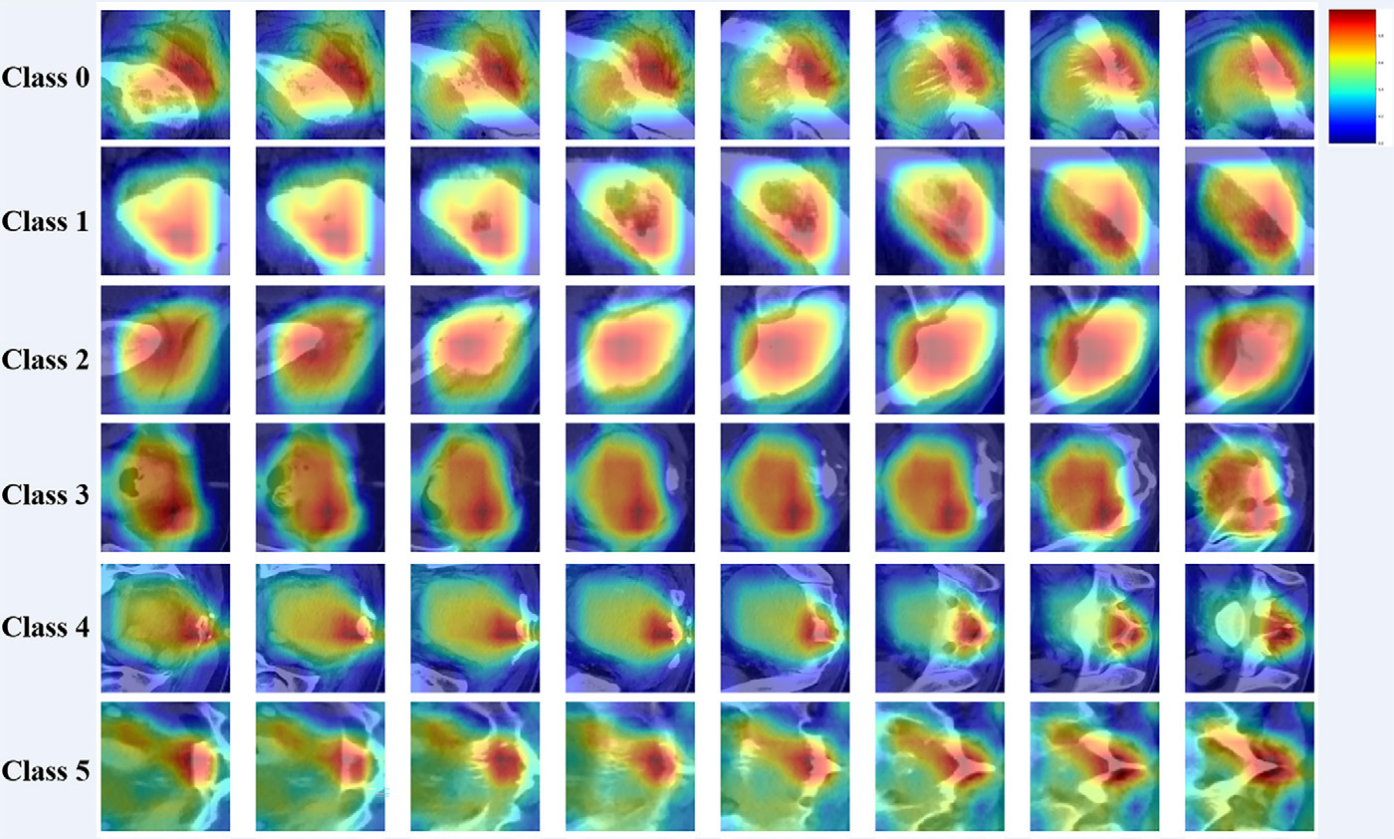
Attention maps of DenseNet121 highlight important regions related to different tumor types. The color scale, from red to blue, represents the increasing contribution of each location to the model’s classification decision.

**Figure 7:**
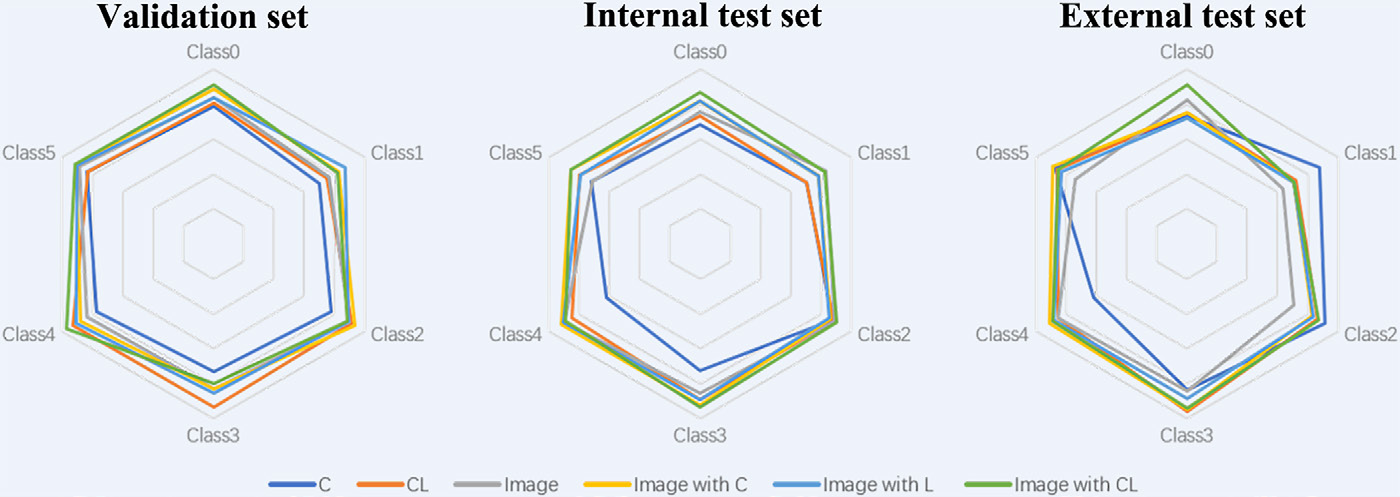
Radar plots show the performance of six different models in distinguishing between six types of tumors. These models are based on clinical data (C), clinical and location data (CL), image, image with C, image with L, and image with CL. The "image with CL" model demonstrates superior overall performance, particularly in the internal test set. The tumors under study are metastatic tumor (class 0), chondrosarcoma (class 1), osteosarcoma (class 2), chordoma (class 3), neurogenic tumor (class 4), and giant cell tumor (class 5).

**Figure 8:**
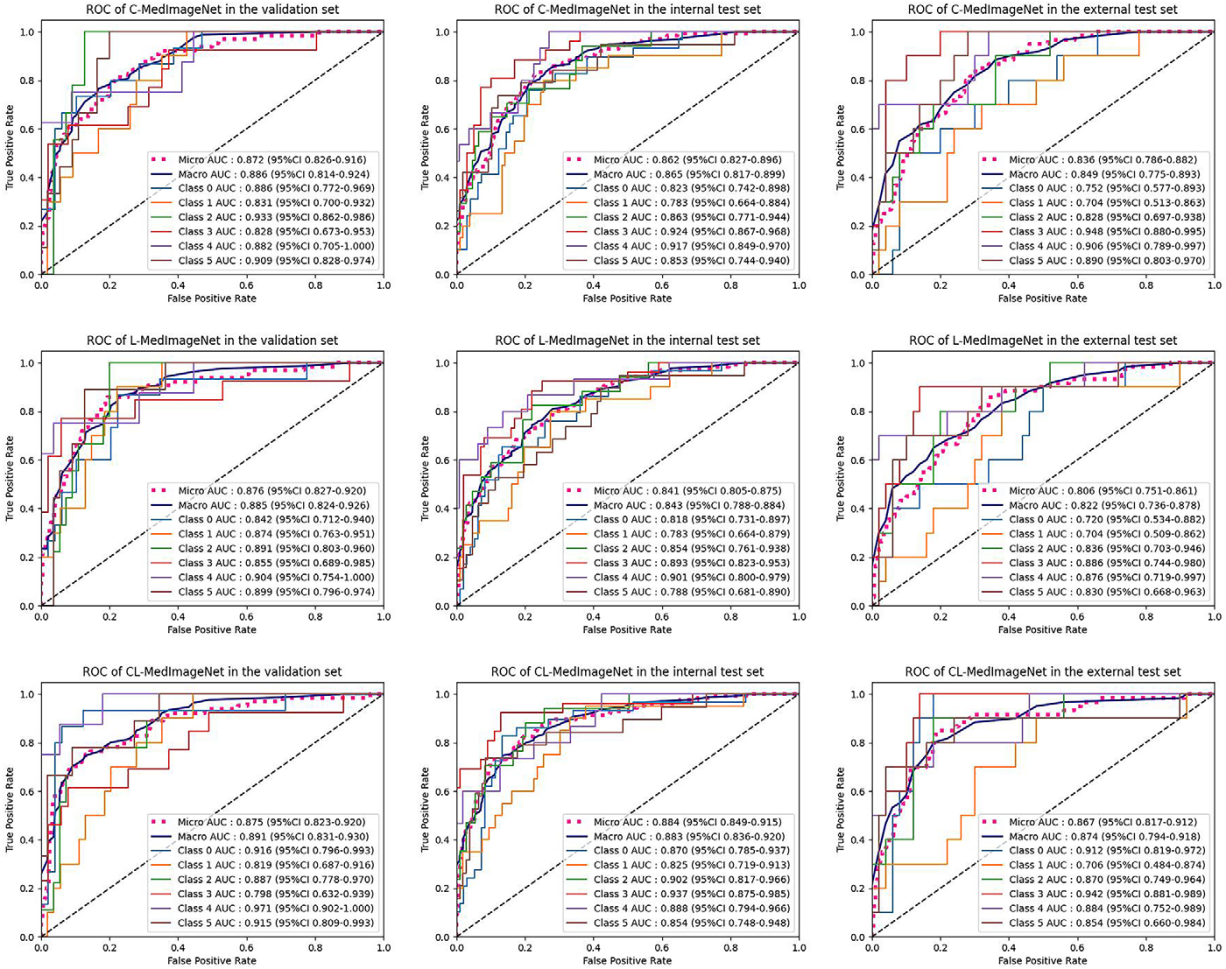
The area under the curve (AUC) values for the C-MedImageNet, L-MedicineNet, and CL-MedicineNet models were plotted for the validation set, internal training set, and external training set. C = clinical, L = location, ROC = receiver operating characteristic.

Additionally, attention maps of DenseNet121 emphasize key regions associated with different tumor types (Fig 6). Both C-MedImageNet and L-MedImageNet outperformed the image-only DenseNet121 model, with C-MedImageNet showing superior performance. It achieved macro average AUC values of 0.89, 0.87, and 0.85 on the validation, internal test, and external test sets, respectively. C-MedImageNet achieved AUC ranges of 0.83 (chordoma) to 0.93 (osteosarcoma), 0.78 (metastatic tumor) to 0.92 (chordoma), and 0.70 (metastatic tumor) to 0.95 (chordoma) across these sets, demonstrating its ability to integrate clinical and image data more effectively than both the SVM model based on clinical information and DenseNet121 (Table S3). CL-MedImageNet demonstrated the best performance, showing macro average AUCs of 0.89 (validation), 0.88 (internal test), and 0.87 (external test), with corresponding macro F1 scores of 0.63, 0.63, and 0.56, respectively. CL-MedImageNet achieved AUC ranges of 0.80 (chordoma) to 0.97 (neurogenic tumor), 0.83 (chondrosarcoma) to 0.94 (chordoma), and 0.71 (chondrosarcoma) to 0.94 (chordoma) on these sets (Fig 7). Detailed classification results for the six sacral tumor types are provided in Table 3 and Figure 8.

**Figure 9:**
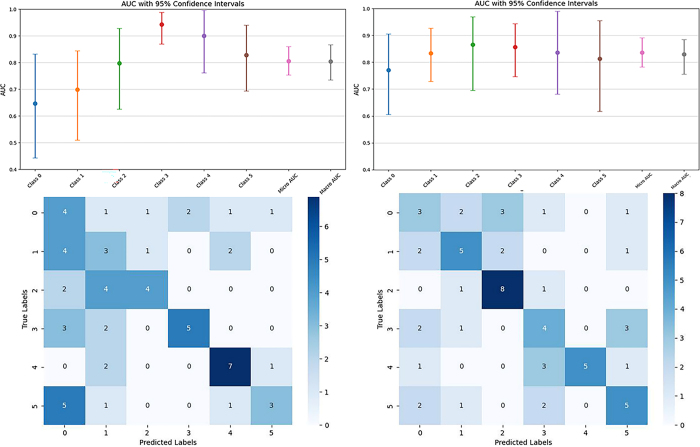
The area under the curve (AUC) values (with 95% CIs) and confusion matrix of the reading results from two radiologists.

The radiologists’ interpretations showed performance comparable to the C-MedImageNet model and the SVM model based solely on clinical data on the external test set, with macro average AUC values of 0.80 and 0.83, respectively (Table S4). The two radiologists achieved AUC ranges of 0.77 (metastatic tumor) to 0.87 (osteosarcoma) and 0.65 (chondrosarcoma) to 0.94 (chordoma) on the external test set (Fig S3). The CL-MedImageNet model outperformed the radiologists’ interpretations (macro average AUCs, 0.87 vs 0.80, *P* = .002; 0.87 vs 0.83, *P* = .45). Detailed classification results for the radiologists are shown in Figure 9 and Table S5.

## Discussion

This study developed an AI-based model to improve the classification of sacral tumors, addressing challenges in late diagnosis and variable treatment responses. A multimodal approach integrating clinical, imaging, and location data (CL-MedImageNet) outperformed other models, with macro AUCs of 0.89, 0.88, and 0.87 across validation and test sets, excelling in identifying neurogenic tumors (AUC, 0.97) and chordomas (AUC, 0.94). The model surpassed radiologists’ performance (0.87 vs 0.80, *P* = .002; 0.87 vs 0.83, *P* = .45), particularly in challenging cases like metastatic tumor (0.91 vs 0.65, *P* < .001; 0.91 vs 0.77,* P* < .001). These results demonstrate that AI-driven multimodal analysis enhances diagnostic accuracy, offering a noninvasive alternative to biopsy and supporting personalized treatment strategies for sacral tumors.

Our study expands the previous research that aimed to predict sacral tumor types before surgery. A study included 95 patients for sacral chordoma and giant cell tumor identification using enhanced CT, where least absolute shrinkage and selection operator plus a classifying generalized linear model showed superior performance with an AUC of 0.98 and an accuracy of 0.90 (13). The no-new-Net model also showed substantial promise in identifying osteosarcomas and Ewing sarcomas with an AUC of 0.84 (35). These models were not fully automated, which could introduce human error. On the other hand, our model uses a fully automated image segmentation and classification process, reducing human error, increasing efficiency, and improving overall accuracy. Moreover, our research shows that the automated hybrid model not only performs a one-time classification into six categories but also includes segmentation.

A substantial limitation of many of the prior studies is their lack of external validation, which could lead to an overestimation of model performance (13,31–34). The rarity of bone tumors has further aggravated the difficulty of performing external tests, contributing to potential biases in internal validation. Model validity should be confirmed using specialized test sets, since current studies frequently validate models on specific datasets that may not generalize effectively (39–41). Unlike these studies, our research addresses this issue by using an external test set for validation, demonstrating the model’s applicability across different clinical environments and ensuring more reliable results.

In addition, we are, to our knowledge, the first to use the hip bone as a reference framework for tumor localization, enhancing tumor recognition and classification accuracy. Yin et al developed radiomics models using NCCT and clinical features that achieved high diagnostic performance (AUCs, 0.80–0.96) for classifying six sacral tumor types, with accuracy ranging from 0.85 to 0.90 across different tumor categories (32). Similarly, a triple-classification radiomics model using MRI differentiated sacral chordoma, giant cell tumor, and metastatic tumor with an AUC of 0.77 (33). These studies only used simple clinical factors and radiomics features, while our study improves upon this by introducing a novel tumor localization method, which integrates tumor location with clinical data. This innovative approach substantially improves the model’s diagnostic performance by adding spatial reference data to imaging features.

In our image segmentation task, we implemented a patch-based segmentation approach due to the large size of our data (approximately 550 × 550 × 60 voxels). Given the limitations of the NVIDIA RTX A6000 GPU, we could not load the entire image at once, so we divided the image into patches with a size of 288 × 288 × 48 voxels. This method not only allowed us to fit the data into memory but also reduced the overall memory load. In our classification task, inputting the entire image into the model would be impractical due to the large size, leading to excessive GPU resource usage, potential loss of important spatial details during downsampling, and class imbalance, where tumors occupy only a small fraction of the image. Cropping along the pelvis is not feasible because some tumors extend beyond the pelvic boundary. The approach of a tight bounding box was applied to reduce redundant information and focus on the target region. By loading the image and its corresponding label mask, we identified the boundaries of the tumor area using nonzero pixels in the mask and calculated the coordinate along the *x, y*, and *z* dimensions. The image and label were then cropped to retain a tight bounding box encompassing the tumor area, thereby reducing the image size and computational resource demands. This approach enables the model to concentrate on meaningful features, improving both training efficiency and performance. Although tight bounding box cropping simplifies processing, it results in the loss of positional information, which we addressed by developing a tumor location representation method based on the pelvic coordinate system.

Bone tumors vary substantially by age, sex, and tumor characteristics (10). Benign tumors are more common in children and adolescents, while malignant tumors exhibit type-specific age patterns: osteosarcoma peaks in adolescents, young adults, and older adults (>60 years), Ewing sarcoma predominantly affects children and adolescents, and chondrosarcoma is more frequent in individuals over 40 years old (42). Male individuals have a 1.5 times higher incidence than female individuals (43). Clinically, benign tumors tend to be smaller and slow-growing, whereas malignant tumors are typically larger, fast-growing, and associated with greater aggressiveness and worse prognosis (44). The univariate analysis of clinical factors and the ablation study of our research are provided in Table S6. The tumor’s location also plays a key role in diagnosis, as different tumors arise from different regions. For example, sacral chordoma is most commonly found below the S3 vertebra, while sacral giant cell tumor is typically located between S1 and S3, as seen on conventional imaging (13). Sacral chordoma is more likely to occur in the sacrococcygeal region, often along the midline, whereas chondrosarcomas are more often found in lateral locations (45). In our study, the hip bone was used as the reference coordinate system instead of the voxel coordinate system, as the variable scanning areas could not accurately represent the true position relative to the body. Therefore, we included information about the relative position of the tumor center of mass with respect to the hip bone. To streamline data processing and analysis, we converted all data into a unified LPS coordinate system. This standardization simplifies the handling of direction, unit, and axis variations, which reduces the complexity of data processing and the difficulty of coding. The consistent coordinate system is particularly advantageous in machine learning and deep learning applications, as it enhances feature extraction and learning processes, thereby improving prediction accuracy. Moreover, a unified coordinate system is essential for precisely measuring the distance between the tumor centroid and the hip bone.

Our model, CL-MedImageNet, outperformed radiologists, particularly in diagnosing metastatic tumors, effectively compensating for this common clinical diagnostic weakness. Metastatic tumors often represent an exclusionary diagnosis that radiologists find challenging to identify without a comprehensive patient history. Typically, a bone scan is the most common method for detecting bone metastasis and is a standard component of the imaging protocol as recommended by the National Comprehensive Cancer Network guidelines (46). While bone scans are highly sensitive, their major drawback is relatively low specificity, primarily due to poor spatial resolution, accumulation of radiopharmaceuticals in normal skeletal structures, soft tissues or viscera, and uptake due to benign processes. This low specificity poses substantial challenges for physicians when manually analyzing scintigraphic images in clinical nuclear medicine (47). Our study demonstrates that our CNN-based model more effectively identifies metastatic tumors, enhancing diagnostic accuracy. However, our model shows relatively lower accuracy in chondrosarcoma diagnosis. This limitation likely reflects the inherent complexity of chondrosarcoma’s diverse pathologic subtypes—including conventional (central), peripheral (secondary), dedifferentiated, mesenchymal, clear cell, and myxoid variants—which present substantial diagnostic challenges. Particularly, the imaging similarities between dedifferentiated type and osteosarcoma, clear cell type and giant cell tumor, and myxoid type and chordoma create substantial classification difficulties. This contrast highlights both the model’s current limitations in handling rare or complex subtypes and its valuable potential to augment radiologists’ diagnostic capabilities. Moreover, the modest F1 scores (about 0.55) reflect inherent challenges in sacral tumor classification: class imbalance, cross-institutional variability, and overlapping tumor characteristics. The model’s performance matches radiologist accuracy (approximately 0.5), demonstrating clinically meaningful detection despite these constraints.

Our study had several limitations. First, the study’s main limitation is its small external test cohort due to the rarity of sacral tumors, particularly affecting uncommon subtype analysis and statistical reliability. The limited samples per tumor category, while unavoidable, may influence machine learning model robustness and generalizability. Additionally, the retrospective design carries inherent biases, including variations in imaging protocols and clinical documentation across centers. These factors necessitate cautious interpretation while reflecting real-world challenges in studying rare oncologic conditions. Additionally, the integration of multimodality imaging represents a promising direction for CNN model development in the medical field. However, the retrospective nature of our study precluded the possibility of developing combination models that use pathology, CT, and MR images. Finally, excluding patients with postoperative tumor recurrence may limit the generalizability of the model, particularly when dealing with recurrent tumors.

In conclusion, we have successfully developed a deep learning model capable of reliably predicting sacral tumor types through a fully automated process, using NCCT scans, clinical, and location information. This highly reproducible and generalizable AI model enables automated, noninvasive sacral tumor characterization with seamless clinical integration, enhancing diagnostic accuracy (particularly for early lesions), supporting tumor-type differentiation, and facilitating personalized treatment planning to improve patient outcomes. To strengthen the validity of our model, future studies should prioritize larger, multicenter collaborations to assemble more extensive and diverse patient cohorts. We plan to address this critical gap in our ongoing research by actively seeking partnerships with additional institutions to compile a more comprehensive dataset for further validation. In addition, while the current study provides meaningful preliminary validation through our external validation approach, we acknowledge that incorporating more robust validation methods such as bootstrapping could further strengthen the reliability and generalizability of our findings.

## Supplemental Files

Appendix S1, Tables S1-S8, Figures S1-S4

Conflicts of Interest
