# Supplementary material for: Assessing an Automated Noncontrast CT–based Pipeline for Sacral Tumor Classification Using a Hip Bone Reference Frame
Source: Radiol Imaging Cancer. 2026 Jan 2;8(1):e250098. doi: 10.1148/rycan.250098 (PMC12862476; doi:10.1148/rycan.250098)
Supplement: Appendix S1, Tables S1-S8, Figures S1-S4 [file rycan250098suppa1.pdf]

Supplemental materials undergo peer review but are not copyedited, and may include typographical errors.

©RSNA, 2025
10.1148/rycan.250098

# Innovative MedImageNet for Automated Sacral Tumor Localization and Multi-Class Identification of Six Tumor Types in NCCT with a Hip Bone Reference Frame

## Appendix S1

### The Inclusion and Exclusion Criteria for Both the Primary and External Cohorts

The primary cohort from center 1 was utilized for model training, hyperparameter optimization, and internal testing and was divided into a training set, a validation set, and an internal test set. This cohort comprised 630 patients from January 2011 to May 2024. Inclusion criteria encompassed histopathologically confirmed sacral tumors, with patients having preoperative NCCT images of single sacral tumors. In clinical practice, multiple metastases are usually easier to diagnose, whereas solitary metastatic tumors can be difficult to diagnose in the absence of relevant clinical history. Therefore, our study included only solitary lesions, which allows for better guidance in clinical practice. Exclusion criteria included prior anticancer treatment, inadequate image quality, repeated patients for follow-up or monitoring, and postoperative tumor recurrence. Additionally, we collected data for an external test cohort from centers 2 and 3, spanning from January 1, 2019, to May 31, 2024, specifically for the final model evaluation. When gathering data from two external centers, we set a minimum requirement of five cases per tumor type at each center. Although some tumor types had six to seven cases at a single center, our focus during the data collection process was on ensuring the representativeness and quality of the data, rather than merely aiming for a large sample size for each tumor type. To ensure the model could be reasonably validated across different tumor types, we ultimately decided to collect 10 cases per class. This balanced approach was intended to ensure that all tumor types were adequately represented, preventing any bias in the model's performance evaluation due to an insufficient number of samples from certain categories. This cohort consisted of 60 patients with sacral tumors, and the inclusion and exclusion criteria remained consistent with those described earlier. Our study included sacral metastases of diverse primary origins (e.g., prostate, breast, lung, renal, and thyroid cancers, as well as

Supplemental materials undergo peer review but are not copyedited, and may include typographical errors.

myeloma) and encompassed all radiographic lesion types: lytic, blastic, and mixed patterns (Table S2). These variations were intentionally incorporated to enhance clinical applicability, with representative examples provided in Figure S3.

**The Scanning Parameters for the Axial NCCT of Each CT Scanner**

All CT images were obtained using multidetector row CT systems (Philips iCT 25, Philips Medical Systems; GE Lightspeed VCT 64, GE Medical Systems). The scanning parameters were as follows: 120 kV, 100-370 mAs, section thickness = 5 mm, field of view = 350 × 350 mm, and matrix = 512 × 512 mm. The reconstruction methods were soft tissue and bone kernel algorithms. It is important to note that all the CT images included in our study were taken with soft tissue window settings. Our use of soft tissue window CT (300-500 HU width, 40-60 HU level) was justified by *(a)* better soft tissue mass visualization for tumor assessment[1], *(b)* pilot tests showing superior boundary delineation (Dice +0.15, $P < .001$ vs bone windows), *(c)* alignment with prior bone tumor AI studies, and *(d)* clinical practicality since many tumors are initially detected on routine CT scans lacking bone windows (49–52).

**Reference Standard for Classification Model: Histopathological Diagnosis by Expert Pathologists**

The definitive diagnosis for all tumors was established by histopathological examination of surgical specimens, performed by two board-certified musculoskeletal pathologists with more than 10 years of experience. Discrepancies were resolved through consensus review with a third senior pathologist. This standard was applied uniformly to all cohorts, including the external validation sets.

**Reference Standard for Segmentation Model: Radiologist-Performed Manual Delineation**

The accuracy of automated tumor segmentation (model 1) was quantified using Dice similarity coefficients, with the reference standard being manual segmentation performed independently by two radiologists (with 5 and 10 years of musculoskeletal imaging experience, respectively). To be more specific, a radiologist with 5 years of experience in musculoskeletal imaging manually delineated the tumor regions of

Supplemental materials undergo peer review but are not copyedited, and may include typographical errors.

interest and hip bones on the NCCT images using ITK-SNAP 3.8.0 *(http://www.itksnap.org)*, layer by layer. A second radiologist with 10 years of experience verified and adjusted the accuracy of these contours.

**The Preprocessing of Automatic Segmentation Model**

Before automatic segmentation, the model's generalization ability is enhanced through various data augmentation techniques, including cropping, flipping, rotation, intensity normalization, and the introduction of noise. Specifically, random affine transformations (RandAffined) are applied with a probability of 0.2 to enrich the dataset through rotation and scaling. To increase data diversity, images and their corresponding labels are randomly flipped along different axes (RandFlipd) with a probability of 0.5. Additionally, Gaussian noise (RandGaussianNoised) is randomly added to the images, with a mean value of 0 and a standard deviation of 0.1, to improve the dataset's robustness. Finally, image intensity values are standardized by linearly scaling them to ensure the pixel values fall within a reasonable range: –80, 250 to –1, 1 (ScaleIntensityRanged).

**SVM Algorithm Implementation with Probabilistic Outputs**

The support vector machine (SVM) algorithm represents a supervised learning approach designed for data analysis and pattern recognition (52,53). By constructing an optimal hyperplane in high-dimensional space, SVM maximizes the margin between distinct classes to achieve effective separation. In our implementation (using scikit-learn), we employed a radial basis function kernel with specific parameters (regularization parameter C = 10 and probability estimation enabled) to facilitate probabilistic classification outputs. Probability estimates were obtained using Platt scaling to enable robust AUC calculation. This configuration was selected to balance model complexity with generalization capability while providing probability estimates for each prediction.

Supplemental materials undergo peer review but are not copyedited, and may include typographical errors.

**Table S1**. The Composition of the Six Types of Sacral Tumors Included in Each Cohort of our Study

| Classification | Training set | Validation set | Internal test set | External test set |
|---|---|---|---|---|
| Metastatic tumor (class 0) | 101 | 15 | 29 | 10 |
| Chondrosarcoma (class 1) | 70 | 10 | 20 | 10 |
| Osteosarcoma (class 2) | 62 | 9 | 17 | 10 |
| Chordoma (class 3) | 90 | 13 | 26 | 10 |
| Neurogenic tumor (class 4) | 53 | 8 | 15 | 10 |
| Giant cell tumor (class 5) | 64 | 9 | 19 | 10 |
| All | 440 | 64 | 126 | 60 |

Supplemental materials undergo peer review but are not copyedited, and may include typographical errors.

**Table S2**. The Primary Tumor Types and Corresponding Case Numbers of Sacral Metastases in the Training, Validation, and Test Sets.

| Set | Breast cancer | Prostate cancer | Lung cancer | Renal cell carcinoma | Thyroid cancer | NA |
|---|---|---|---|---|---|---|
| Training set | 28 | 21 | 23 | 7 | 5 | 17 |
| Validation set | 7 | 1 | 3 | 2 | 0 | 2 |
| Internal test set | 11 | 4 | 6 | 3 | 1 | 4 |
| External test set | 3 | 2 | 4 | 0 | 1 | 0 |

Note.—NA = tumors of unknown origin or cases where relevant information was not available.

Supplemental materials undergo peer review but are not copyedited, and may include typographical errors.

**Table S3**. The AUC Values with 95% CIs for Each Category (SVM, DenseNet121, C-MedImageNet, L-MedImageNet, C-MedImageNet) During Validation and Internal/External Testing.

| Model | Class 0 | Class 1 | Class 2 | Class 3 | Class 4 | Class 5 |
|---|---|---|---|---|---|---|
| C-SVM (validation) | 0.788(0.660-0.896) | 0.698(0.520, 0.852) | 0.776(0.598, 0.910) | 0.730(0.579, 0.853) | 0.770(0.532, 0.958) | 0.834(0.717, 0.929) |
| C-SVM (internal test) | 0.688(0.578, 0.788) | 0.703(0.567, 0.829) | 0.873(0.770, 0.953) | 0.725(0.630, 0.825) | 0.620(0.468, 0.755) | 0.718(0.594, 0.834) |
| C-SVM (external test) | 0.732(0.596, 0.857) | 0.876(0.752, 0.963) | 0.914(0.808, 0.995) | 0.838(0.620, 0.972) | 0.616(0.431, 0.817) | 0.872(0.779, 0.951) |
| CL-SVM (validation) | 0.807(0.665, 0.924) | 0.748(0.599, 0.884) | 0.915(0.826, 0.972) | 0.934(0.861, 0.983) | 0.929(0.837, 0.995) | 0.830(0.672, 0.960) |
| CL-SVM (internal test) | 0.735(0.618, 0.833) | 0.707(0.606, 0.799) | 0.883(0.804, 0.944) | 0.893(0.818, 0.950) | 0.845(0.694, 0.966) | 0.794(0.668, 0.905) |
| CL, SVM (external test) | 0.750(0.600, 0.874) | 0.724(0.588, 0.845) | 0.864(0.740, 0.969) | 0.960(0.900, 1.000) | 0.848(0.636, 0.998) | 0.858(0.734, 0.952) |
| DenseNet121 (validation) | 0.841(0.724, 0.943) | 0.765(0.610, 0.891) | 0.899(0.810, 0.967) | 0.828(0.682, 0.947) | 0.839(0.592, 0.992) | 0.887(0.756, 0.976) |
| DenseNet121 (internal test) | 0.757(0.657, 0.850) | 0.835(0.750, 0.915) | 0.891(0.801, 0.957) | 0.857(0.777, 0.922) | 0.900(0.804, 0.978) | 0.711(0.586, 0.837) |
| DenseNet121 (external test) | 0.828(0.673, 0.960) | 0.634(0.466, 0.785) | 0.706(0.482, 0.905) | 0.844(0.719, 0.957) | 0.850(0.686, 0.984) | 0.738(0.549, 0.898) |

Supplemental materials undergo peer review but are not copyedited, and may include typographical errors.

| | | | | | | |
|---|---|---|---|---|---|---|
| C-MedImageNet (validation) | 0.886(0.772, 0.969) | 0.831(0.700, 0.932) | 0.933(0.862, 0.986) | 0.828(0.673, 0.953) | 0.882(0.705, 1.000) | 0.909(0.828, 0.974) |
| C-MedImageNet (internal test) | 0.823(0.742, 0.898) | 0.783(0.664, 0.884) | 0.863(0.771, 0.944) | 0.924(0.867, 0.968) | 0.917(0.849, 0.970) | 0.853(0.744, 0.940) |
| C-MedImageNet (external test) | 0.752(0.577, 0.893) | 0.704(0.513, 0.863) | 0.828(0.697, 0.938) | 0.948(0.880, 0.995) | 0.906(0.789, 0.997) | 0.890(0.803, 0.970) |
| L-MedImageNet (validation) | 0.842(0.712, 0.940) | 0.874(0.763, 0.951) | 0.891(0.803, 0.960) | 0.855(0.689, 0.985) | 0.904(0.754, 1.000) | 0.899(0.796, 0.974) |
| L-MedImageNet (internal test) | 0.818(0.731, 0.897) | 0.783(0.664, 0.879) | 0.854(0.761, 0.938) | 0.893(0.823, 0.953) | 0.901(0.800, 0.979) | 0.788(0.681, 0.890) |
| L-MedImageNet (external test) | 0.720(0.534, 0.882) | 0.704(0.509, 0.862) | 0.836(0.703, 0.946) | 0.886(0.744, 0.980) | 0.876(0.719, 0.997) | 0.830(0.668, 0.963) |
| CL-MedImageNet (validation) | 0.916(0.796, 0.993) | 0.819(0.687, 0.916) | 0.887(0.778, 0.970) | 0.798(0.632, 0.939) | 0.971(0.902, 1.000) | 0.915(0.809, 0.993) |
| CL-MedImageNet (internal test) | 0.870(0.785, 0.937) | 0.825(0.719, 0.913) | 0.902(0.817, 0.966) | 0.937(0.875, 0.985) | 0.888(0.794, 0.966) | 0.854(0.748, 0.948) |
| CL-MedImageNet (external test) | 0.912(0.819, 0.972) | 0.706(0.484, 0.874) | 0.870(0.749, 0.964) | 0.942(0.881, 0.989) | 0.884(0.752, 0.989) | 0.854(0.660, 0.984) |

Note: C = clinical information; L = location information. Class 0, class 1, class 2, class 3, class 4, and class 5 respectively stand for metastatic tumor, chondrosarcoma, osteosarcoma, chordoma, neurogenic tumor, and giant cell tumor.

Supplemental materials undergo peer review but are not copyedited, and may include typographical errors.

**Table S4**. AUC values and F1 scores with 95% CIs for radiologist used to identify various types of sacral tumors in the external training set.

| | External test set | | | |
|---|---|---|---|---|
| | Macro average | | Micro average | |
| | AUC | F1 score | AUC | F1 score |
| Radiologist 1 Interpretation | 0.804 (0.735, 0.867) | 0.450 (0.316, 0.563) | 0.806 (0.754, 0.860) | 0.433 (0.317, 0.567) |
| Radiologist 2 Interpretation | 0.830 (0.756, 0.885) | 0.503 (0.359, 0.618) | 0.837 (0.782, 0.891) | 0.500 (0.367, 0.633) |

Supplemental materials undergo peer review but are not copyedited, and may include typographical errors.

**Table S5**. Diagnostic Performance Comparison: CL-Medimagenet versus Radiologists across Tumor Types (AUC Values with 95% CIs)

| | Class 0 | Class 1 | Class 2 | Class 3 | Class 4 | Class 5 |
|---|---|---|---|---|---|---|
| Radiologist 1 Interpretation | 0.647(0.443, 0.832) | 0.699(0.509, 0.844) | 0.797(0.625, 0.929) | 0.943(0.870, 0.989) | 0.901(0.761, 0.997) | 0.829(0.694, 0.941) |
| Radiologist 2 Interpretation | 0.771(0.606, 0.905) | 0.834(0.729, 0.927) | 0.866(0.695, 0.970) | 0.857(0.747, 0.944) | 0.836(0.682, 0.990) | 0.813(0.617, 0.956) |
| CL-MedImageNet | 0.912(0.819, 0.972) | 0.706(0.484, 0.874) | 0.870(0.749, 0.964) | 0.942(0.881, 0.989) | 0.884(0.752, 0.989) | 0.854(0.660, 0.984) |

Note.—Class 0, class 1, class 2, class 3, class 4, and class 5 respectively stand for metastatic tumor, chondrosarcoma, osteosarcoma, chordoma, neurogenic tumor, and giant cell tumor. C = clinical information; L = location information.

Supplemental materials undergo peer review but are not copyedited, and may include typographical errors.

**Table S6**. Univariate Analysis and Ablation Studies Were Performed Individually Based on Sex, Age, and Tumor Volume Using SVM.

| Method | Validation Set | | | | Internal Test Set | | | | External Test Set | | | |
|---|---|---|---|---|---|---|---|---|---|---|---|---|
| | Macro Average | | Micro Average | | Macro Average | | Micro Average | | Macro Average | | Micro Average | |
| | AUC | F1 Score | AUC | F1 Score | AUC | F1 Score | AUC | F1 Score | AUC | F1 Score | AUC | F1 Score |
| Sex | 0.540 (0.469-0.606) | 0.115 (0.068-0.156) | 0.578 (0.502-0.656) | 0.250 (0.156-0.359) | 0.534 (0.484-0.587) | 0.120 (0.086-0.152) | 0.591 (0.538-0.648) | 0.260 (0.181-0.339) | 0.515 (0.438-0.589) | 0.090 (0.045-0.136) | 0.518 (0.433-0.600) | 0.180 (0.082-0.279) |
| Age | 0.750 (0.680-0.814) | 0.240 (0.147-0.320) | 0.760 (0.701-0.818) | 0.297 (0.203-0.422) | 0.689 (0.634-0.743) | 0.265 (0.191-0.330) | 0.734 (0.692-0.777) | 0.315 (0.244-0.402) | 0.647 (0.563-0.723) | 0.241 (0.142-0.349) | 0.669 (0.595-0.741) | 0.279 (0.164-0.393) |
| Volume | 0.590 (0.491-0.670) | 0.195 (0.125-0.261) | 0.627 (0.546-0.705) | 0.344 (0.234-0.453) | 0.560 (0.500-0.610) | 0.097 (0.066-0.130) | 0.611 (0.560-0.662) | 0.189 (0.126-0.252) | 0.622 (0.532-0.683) | 0.096 (0.050-0.152) | 0.575 (0.500-0.652) | 0.180 (0.082-0.279) |
| Sex and Age | 0.770 (0.710-0.827) | 0.276 (0.179-0.366) | 0.772 (0.713-0.824) | 0.328 (0.219-0.453) | 0.708 (0.658-0.759) | 0.302 (0.219-0.372) | 0.747 (0.704-0.788) | 0.339 (0.260-0.433) | 0.664 (0.584-0.733) | 0.234 (0.130-0.334) | 0.685 (0.618-0.756) | 0.262 (0.164-0.377) |
| Sex and Volume | 0.602 (0.507-0.681) | 0.137 (0.071-0.197) | 0.593 (0.516-0.668) | 0.234 (0.125-0.344) | 0.589 (0.529-0.638) | 0.150 (0.098-0.201) | 0.608 (0.556-0.660) | 0.220 (0.150-0.299) | 0.630 (0.542-0.694) | 0.131 (0.057-0.204) | 0.579 (0.499-0.658) | 0.180 (0.082-0.279) |
| Age and Volume | 0.735 (0.649-0.794) | 0.299 (0.191-0.385) | 0.758 (0.698-0.821) | 0.359 (0.250-0.484) | 0.719 (0.656-0.767) | 0.291 (0.214-0.356) | 0.741 (0.697-0.786) | 0.315 (0.244-0.394) | 0.651 (0.550-0.722) | 0.244 (0.147-0.329) | 0.655 (0.581-0.726) | 0.262 (0.164-0.377) |

Supplemental materials undergo peer review but are not copyedited, and may include typographical errors.

**Table S7**. The Precision and Sensitivity for Each Category (SVM, DenseNet121, C-MedImageNet, L-MedImageNet, C-MedImageNet) During Validation and Internal/External Testing

| | C-SVM | CL-SVM | Densnet121 | C-MedImageNet | L-MedImageNet | CL-MedImageNet |
|---|---|---|---|---|---|---|
| Macro average precision (validation set) | 0.351 | 0.542 | 0.548 | 0.608 | 0.585 | 0.640 |
| Macro average sensitivity (validation set) | 0.341 | 0.528 | 0.458 | 0.609 | 0.598 | 0.643 |
| Micro average precision (validation set) | 0.344 | 0.531 | 0.469 | 0.609 | 0.578 | 0.656 |
| Micro average sensitivity (validation set) | 0.344 | 0.531 | 0.469 | 0.609 | 0.578 | 0.656 |
| Macro average precision (internal test set) | 0.317 | 0.532 | 0.527 | 0.493 | 0.516 | 0.642 |
| Macro average sensitivity (internal test set) | 0.354 | 0.494 | 0.488 | 0.494 | 0.513 | 0.627 |
| Micro average precision (internal test set) | 0.354 | 0.512 | 0.492 | 0.508 | 0.524 | 0.643 |
| Micro average sensitivity (internal test set) | 0.354 | 0.512 | 0.492 | 0.508 | 0.524 | 0.643 |
| Macro average precision (external test set) | 0.492 | 0.473 | 0.375 | 0.514 | 0.448 | 0.564 |
| Macro average sensitivity (external test set) | 0.482 | 0.461 | 0.383 | 0.500 | 0.417 | 0.550 |
| Micro average precision (external test sett) | 0.483 | 0.467 | 0.383 | 0.500 | 0.417 | 0.550 |
| Micro average sensitivity (external test set) | 0.483 | 0.467 | 0.383 | 0.500 | 0.417 | 0.550 |

Note: C = clinical information, L = location information.

Supplemental materials undergo peer review but are not copyedited, and may include typographical errors.

**Table S8**. The Precision and Sensitivity for the Two Radiologists During Validation And Internal/External Testing

| | Radiologist 1 | Radiologist 2 |
|---|---|---|
| Macro average precision (external test set) | 0.512 | 0.539 |
| Macro average sensitivity (external test set) | 0.433 | 0.500 |
| Micro average precision (external test set) | 0.433 | 0.500 |
| Micro average sensitivity (external test set) | 0.433 | 0.500 |

Supplemental materials undergo peer review but are not copyedited, and may include typographical errors.

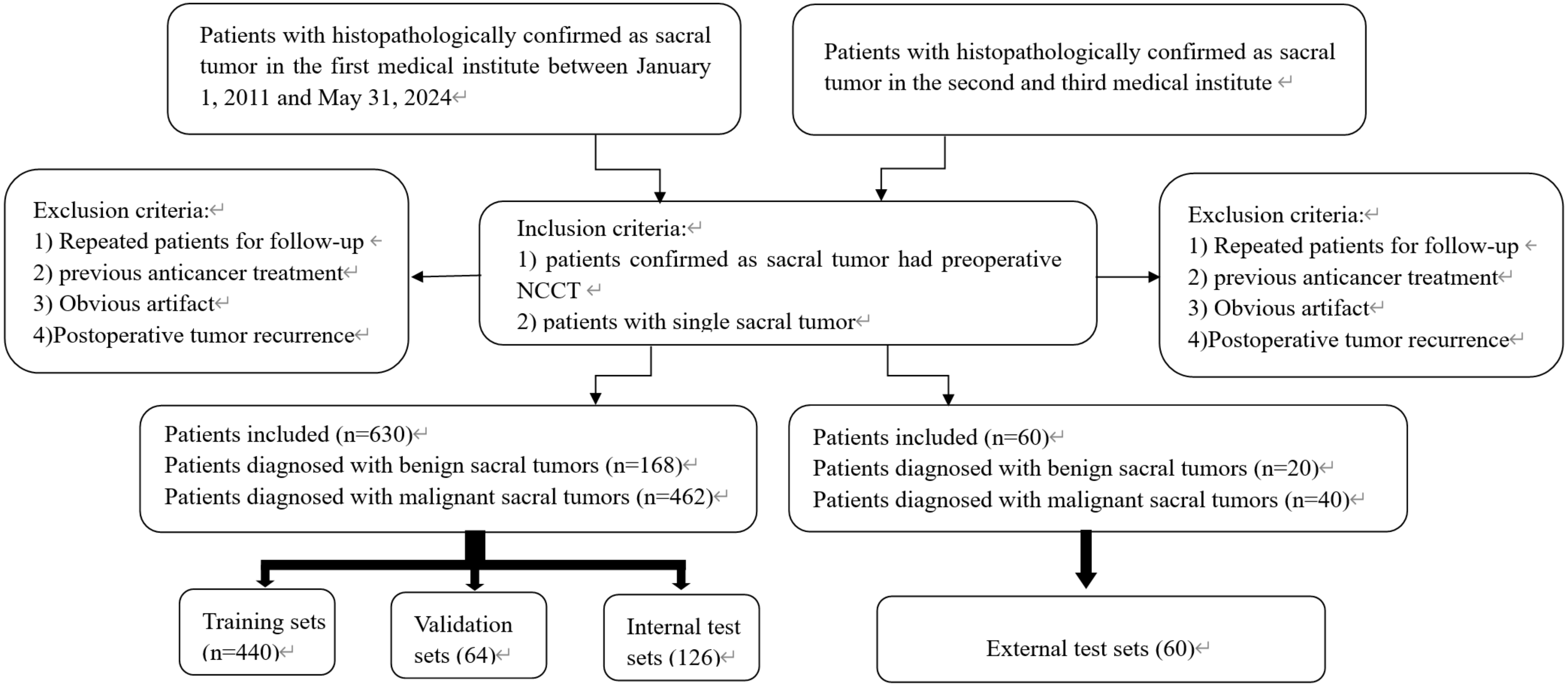


**Figure S1**. Patient selection workflow diagram.

Supplemental materials undergo peer review but are not copyedited, and may include typographical errors.

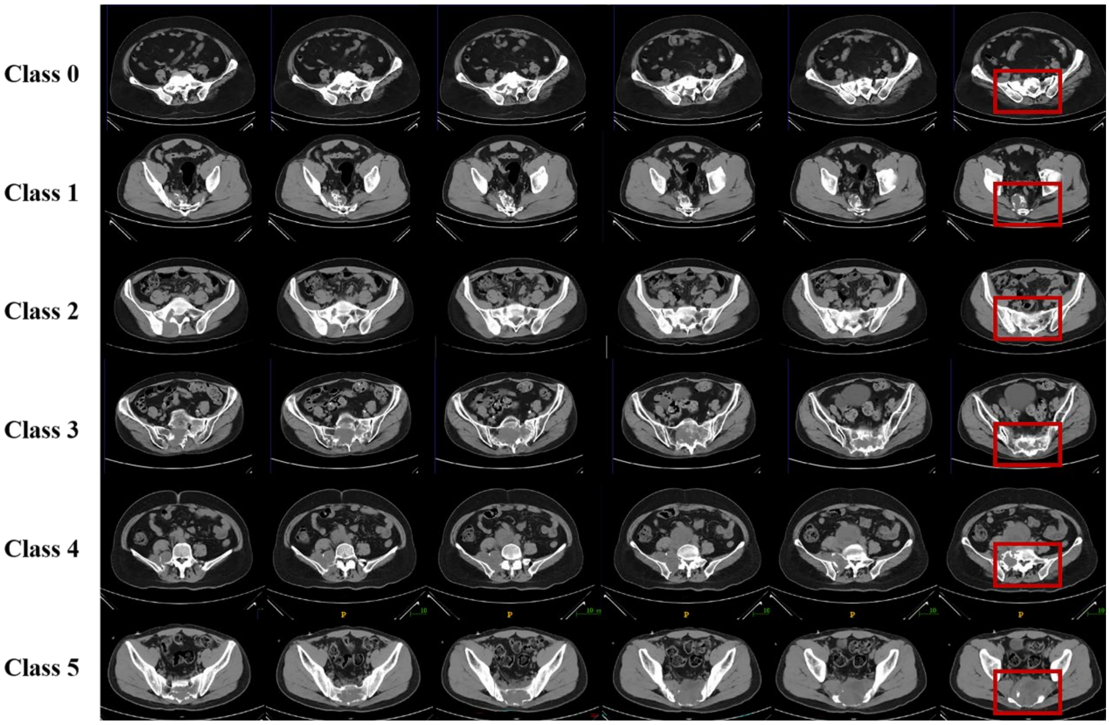


**Figure S2:** Presents a typical example of each tumor type, with the tumor regions highlighted in red boxes. From top to bottom, the classes include class 0 (metastatic tumor), class 1 (chondrosarcoma), class 2 (osteosarcoma), class 3 (chordoma), class 4 (neurogenic tumor), and class 5 (giant cell tumor). The red boxes indicate the areas where the tumors are located on the CT images.

Supplemental materials undergo peer review but are not copyedited, and may include typographical errors.

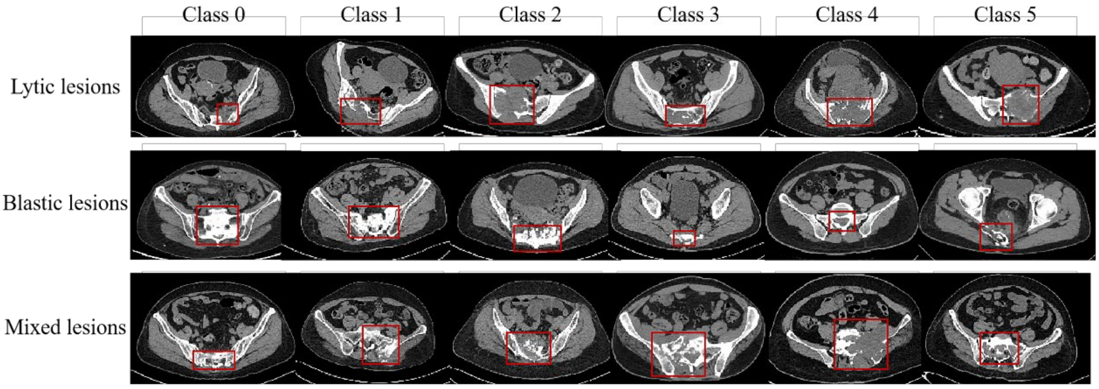


**Figure S3:** Representative examples of each tumor type: lytic, blastic, and mixed lesions. From left to right, the classes include class 0 (metastatic tumor), class 1 (chondrosarcoma), class 2 (osteosarcoma), class 3 (chordoma), class 4 (neurogenic tumor), and class 5 (giant cell tumor). The red boxes indicate the areas where the tumors are located on the CT images.

Supplemental materials undergo peer review but are not copyedited, and may include typographical errors.

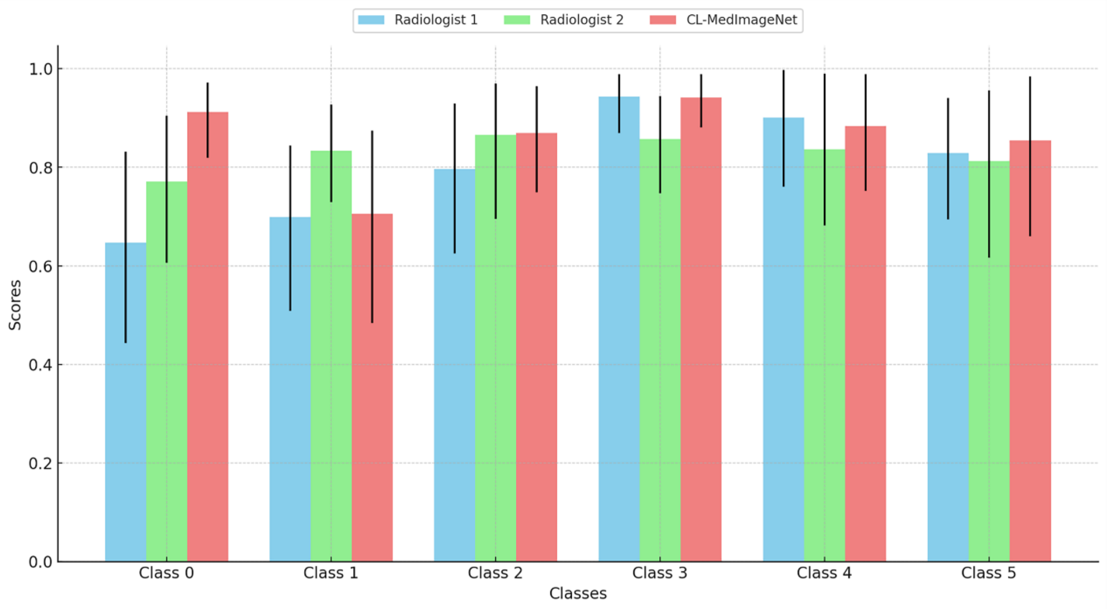


**Figure S4:** Comparison of performance between CL-MedImageNet and radiologists in different tumor types, showing AUC values and their 95% CIs. From left to right, the classes include class 0 (metastatic tumor), class 1 (chondrosarcoma), class 2 (osteosarcoma), class 3 (chordoma), class 4 (neurogenic tumor), and class 5 (giant cell tumor).

Supplemental materials undergo peer review but are not copyedited, and may include typographical errors.

## Reference


48. Kobayashi H, Makise N, Ushiku T, et al. Infiltrative nature of tumor-induced osteomalacia lesions in bone: Correlation between radiological and histopathological features. *Journal of orthopaedic science : official journal of the Japanese Orthopaedic Association.* 2019;24(5):900-905.
49. Yin P, Sun C, Wang S, Chen L, Hong N. Clinical-Deep Neural Network and Clinical-Radiomics Nomograms for Predicting the Intraoperative Massive Blood Loss of Pelvic and Sacral Tumors. *Frontiers in oncology.* 2021;11:752672.
50. Chen W, Ayoub M, Liao M, et al. A fusion of VGG-16 and ViT models for improving bone tumor classification in computed tomography. *Journal of bone oncology.* 2023;43:100508.
51. Nie P, Zhao X, Ma J, et al. Can the preoperative CT-based deep learning radiomics model predict histologic grade and prognosis of chondrosarcoma? *European journal of radiology.* 2024;181:111719.
52. Nedaie A, Najafi AA. Support vector machine with Dirichlet feature mapping. *Neural networks : the official journal of the International Neural Network Society.* 2018;98:87-101.
53. Kumari A, Akhtar M, Shah R, Tanveer M. Support matrix machine: A review. *Neural networks : the official journal of the International Neural Network Society.* 2025;181:106767.
